# Evaluation of Suicide Attempt due to Drug Poisoning in a 7-Year-Old Girl: A Case Report

**Published:** 2020-04

**Authors:** Aliashraf Mozafari, Ali Sahebi, Amir Adibi, Mohammad Saatchi, Kourosh Sayehmiri

**Affiliations:** 1 Department of Epidemiology and Biostatistics, School of Public Health, Tehran University of Medical Sciences, Tehran, Iran.; 2 Clinical Research Development Unit, Shahid Mustafa Khomeini Hospital, Ilam University of Medical Sciences, Ilam, Iran.; 3 Department of Child and Adolescent Psychiatry, Ilam University of Medical Sciences, Ilam, Iran.; 4 Biostatistics Department, School of Health, Ilam University of Medical Sciences, Ilam, Iran.

**Keywords:** *Case Report*, *Child Behavior Checklist (CBCL)*, *Rug Poisoning*, *Suicide Attempt*

## Abstract

**Introduction: **Suicidal behavior is a worrying issue in children and is a very important indicator of emotional distress in children. Suicide is uncommon in children before puberty.

**Case Report:** Here, a case of a 7-year-old girl who committed suicide by drug poisoning was reported. The child’s behavior was assessed using a child behavior checklist (CBCL), in which aggressive behavioral problem was the most important. Her mother had a history of suicide attempts, generalized anxiety, and major depressive disorder over the past year, and her father was a drug abuser.

**Conclusion: **Mental disorders in parents and tensions in the family may be associated with behavioral-emotional difficulties in children and it can lead to dangerous behaviors such as suicide attempts. It is most important to describe the factors that lead to suicide attempt among children and intervention that my help these children.

A suicide attempt is one of the most important indicators of mental health in different communities (1). This worrying phenomenon, with an increasing trend, occurs in all age groups in communities, although most of its occurrences are observed in 15-30 years age group (2). Suicide attempts are less frequently reported in children under the age of 10 years, because most studies on suicidal behaviors usually exclude this age group, thus, the epidemiology and risk factors of suicide behaviors remain unknown in this age group (3). 

 Worldwide annual suicide rates in 5-14 years age group are 0.5/100 000 for boys and 0.9/100 000 for girls (4). In the United States, a total of 14 852 children in the age group of 5-11 years were hospitalized due to suicidal thinking or suicide attempt from 2008 to 2015. Most of them have been associated with an increased prevalence of behavioral problems, especially mood and depression disorders (5, 6). In Iran, the prevalence of psychological disorders in 6-11-year-old children in Tehran is estimated to be approximately 17.9% (7). 

This study aimed to report a case of suicide attempt in a 7-year-old girl and to clarify the family background and emotional-behavioral problems in this child and its relation to the suicide attempt.

## Case Report

The patient was a 7-year-old girl, who was taken to the Emergency Ward of Imam Khomeini hospital in Ilam, southwest of Iran, by her parents. On admission, the vital signs of the patient were checked (BP = 103/68, RR = 18, T = 37.1, Spo2 = 94%, PR =116, GCS = 10/15). She had a decreased level of consciousness but was not cyanotic. 

The patient’s mother noted that the patient had taken more than 25 alprazolam 0.5 tablets. Emergency procedures and gastric lavage were performed to remove pills from the stomach. The patient was transferred to the intensive care unit. After 24 hours, the patient was transferred to the pediatric ward with elevated consciousness and normal vital sign. 

In the pediatric ward, the patient was able to verbally communicate and started out giving answers to the questions asked by the social worker. Based on her medical history, she has not had any evidence of diseases. The patient stated that she took some pills to commit suicide because of her father’s abusive behavior towards her mother. After complete recovery, the patient was discharged from the hospital and referred to the Welfare Support Center.

At the supportive center, the patient's mother was invited to participate in an interview about her family and her daughter's behavioral characteristics. The patient's mother accepted to be interviewed and thus informed consent was obtained from her. The mother was 21 years old, with elementary education, and was under psychiatric drug treatment. She had a history of major depressive disorder, general anxiety, and suicide attempt in the past year. She has been abused by her husband several times. The father was 25 years old with high school education. He was an opium addict and unemployed. Their family social and economic class was low, and they lived in a rented house on the outskirts of the town. Finally, the mother filled the Child Behavior Checklist (CBCL Questionnaire). The patient underwent supportive psychotherapy and now lives with her paternal grandmother

## Discussion

By the use of the CBCL behavioral disorders Checklist, this study aimed to determine behavioral difficulties in a 7-year-old girl who attempted suicide.

CBCL 6-18 is completed by parents or other individuals who know the child well. It obtains parent's reports of children's competencies and behavioral problems. 

The CBCL/6-18 is to be used with children aged 6-18 years. The Iranian version of this questionnaire consists of 113 questions, scored on a 3-point Likert scale (0 = absent, 1 = sometimes, 2 = often). The time frame for item responses is the past 6 months. The 2001 revision of the CBCL/6-18 is made of 8 syndrome scale: anxious/depressed, depressed, somatic complaints, social problems, though problems, attention problems, rule-breaking behavior, and aggressive behavior. 

 CBCL measures 3 broadband scores: (1) internalizing behavior disorder; (2) externalizing behavior disorder; and (3) total problems. Internal consistency (coefficient of Cronbach) was 0.91, which showed high reliability. CBCL is a well-known questionnaire worldwide (8).

The results of the analysis of the CBCL questionnaire showed that this child had high scores in aggressive behavior and social problems. Thought, attention, anxiety, and depression problems were in the next ranks. The lowest scores were related to the behavior of delinquent law-breaking and somatic complaints. In broadband scores of the questionnaire, externalizing behavior disorder was dominant. Suicide attempts are reported less in children under 10 years and usually are due to specific emotional disorders in children (3). Aggressive behavior disorder, which is based on obtained scores, was the predominant behavioral problem in this child.

 In this case, her father was an opium addict, her mother had a history of major depression disorder, general anxiety disorder and suicide attempt in the past year, and there have been tensions in the family during the past year. Thus, it may be concluded that the existence of behavioral-emotional difficulties, such as aggressiveness in this child, was affected by psychiatric disorders in parents and tensions in the family, which led to dangerous behaviors, such as suicide attempt. This lends support to previous findings in the literature that indicated mental disorders, in parents, such as depression, are associated with a higher risk of emotional-behavioral difficulties in children (9, 10). There is strong relationship between suicide attempts and aggression disorder (4, 11). Our study provided additional support for this relationship. The family's socioeconomic level was low, which might have caused tensions in the family. In previous studies, low socioeconomic status of parents was associated with children’s psychopathology (12). However, it can be concluded that the interaction between these risk factors has led to risky behaviors, such as suicide attempts, in this child. Social, emotional, and behavioral problems are temporary for most children. The problems are often resolved through guidance and support from family. If these difficulties continue or worsen, the child may develop a disorder or risk of some type of mental disorder (13). 

Childhood emotional-behavioral problems have significant negative impacts on the individual, family, and society. With increase in age, these disorders become very difficult to treat, so early intervention and prevention of these disorders during childhood can improve developmental and social outcomes later in childhood.
